# Transient myoclonic state or transient myoclonic state with asterixis: A systematic review

**DOI:** 10.3892/mi.2025.228

**Published:** 2025-03-19

**Authors:** Jamir Pitton Rissardo, Nilofar Murtaza Vora, Nirali Seth, Sanobar Shariff, Ana Letícia Fornari Caprara

**Affiliations:** 1 Department of Neurology, Cooper University Hospital, Camden, NJ 08103, USA; 2 Department of Medicine, Terna Speciality Hospital, Navi Mumbai 400706, India; 3 Department of Medicine, Lady Hardinge Medical College, Delhi 110001, India; 4 Department of Medicine, Yerevan State Medical University, Yerevan 0025, Armenia

**Keywords:** transient myoclonic state, myoclonus, transient myoclonic state with asterixis, transient co-occurrence of myoclonus and asterixis

## Abstract

Transient myoclonic state (TMS) is a rare type of myoclonic jerks occurring predominantly in the upper extremities involving the head and commonly associated with asterixis. The present study performed a systematic review of published articles on this condition. For this purpose, six databases were searched by two reviewers to identify reports on TMS published online until November, 2024. A total of 17 reports containing 78 cases were found. Almost all the reports were from Japan, apart from one case reported in the USA. The mean age of the patients was 75.67 years (standard deviation, 5.8 years) and the median age was 75 years (range, 54 to 84 years). Sex was reported in 74 reports, and 60.8% of the patients were males. A precipitating factor, such as an infectious disease or the introduction of a new medication was observed in 24 cases (30.7%). All individuals achieved full recovery; however, 53 patients (67.9%) required benzodiazepine therapy, while the remaining individuals improved spontaneously. In summary, the present systematic review demonstrates that TMS is a rare condition, and is mainly encountered in Japan by unknown factors. There are likely genetic and environmental factors involved in its development; however, no specific geolocation related to the occurrence of TMS in Japan was found. It has a benign course and usually improves with the prescription of benzodiazepines. Management strategies include ensuring adequate multidisciplinary care coordination, as well as educating patients and their families about TMS. Future studies are required to describe the cases of TMS, including videos of the phenomenology. It is also recommended to perform whole genome sequencing analysis in patients with TMS.

## Introduction

In 1992, Hashimoto *et al* (1) coined the term transient myoclonic state with asterixis (TMA) in a group of older Japanese individuals presenting with myoclonus and asterixis occurring at the same time. In this context, Mizutani *et al* (2) were the first to describe in the literature a similar stereotyped myoclonus. Notably, the patient reported by Mizutani *et al* (2) had positive serum Epstein-Barr virus antibodies, which was likely a coincidental finding. Following the reports by Hashimoto *et al* (1) and Mizutani *et al* (2), other patients with transient myoclonic state (TMS) and no history of asterixis were reported (3). In this context, TMA and TMS represent different conditions within the same spectrum, which are hereby collectively referred to as TMS.

Myoclonus has a hyperkinetic phenomenology defined by jerk-like movements that occur secondary to neuronal discharges (4). Myoclonus can either present as physiological jerks or may be associated with some underlying neurodegenerative disorder (5). It can originate in the cortex, brainstem, spinal cord or peripheral nerves. Myoclonus usually persists for a number of years without any identifiable physiological cause (6). Positive myoclonus are jerks caused by the activation of a determined group of muscles, whereas negative myoclonus, also known as asterixis, are jerks that occur due to the cessation of ongoing muscular activity (4). In addition, positive myoclonus occurs more commonly, and negative myoclonus occurs mainly in hospital settings (6).

A small number of patients have presented with TMS, considered a unique type of myoclonic jerk, as it primarily involves faciobrachial structures and is characterized by repetitive and irregular movements. However, lower extremity involvement has been reported in ~50% of cases (7). A single jerk consisted of a burst of myoclonic muscle contractions and lasted for a few seconds. These bursts of myoclonic contractions can be combined with asterixis-like movements in some patients. TMS is most commonly reported amongst older patients concomitant with comorbidities, such as chronic kidney injury, diabetes mellitus and hypertension. These patients, however, are independently carrying out their activities of daily living.

A key concern regarding TMS is the limited understanding of its condition definition (7). TMS presents with classic clinical symptoms of repetitive bilateral myoclonus lasting for a few seconds with consciousness fully intact. The syndrome has a benign prognosis and is usually self-limiting. Despite such characteristic presentation, the limited knowledge of TMS can lead to the misdiagnosis of the condition as metabolic encephalopathy or epilepsy (3). Furthermore, frequent recurrence of the syndrome has been reported. Therefore, the timely diagnosis of the condition is of utmost importance so that proper management can be provided to the patients (8). The present systematic review aimed to provide critical insight and up-to-date information on the latest information about the pathophysiology and therapy of TMS.

## Data and methods

### Study selection

A comprehensive search was conducted across six major databases to identify all available reports on TMS, published in electronic format up to November, 2024. The databases, Latin American and Caribbean Health Sciences Literature (Lilacs) (https://lilacs.bvsalud.org/en/), Excerpta Medica (Embase) (https://www.embase.com/), PubMed (https://pubmed.ncbi.nlm.nih.gov/), Google Scholar (https://scholar.google.com/), Scientific Electronic Library Online (Scielo) (https://www.scielo.org/), ScienceDirect (https://www.sciencedirect.com/) and CiNii Japan Science and Technology Agency (https://cir.nii.ac.jp/) were searched. The search terms used were ‘transient myoclonus’, ‘transient myoclonus state’, ‘transient myoclonus state with asterixis’, ‘positive and negative myoclonus’ (Table I).

### Inclusion and exclusion criteria

All types of articles, including reports on TMS, were included. No language restrictions were applied; for manuscripts that did not provide sufficient information in English, Google Translate was used (9).

### Study selection

**Figure 1:**
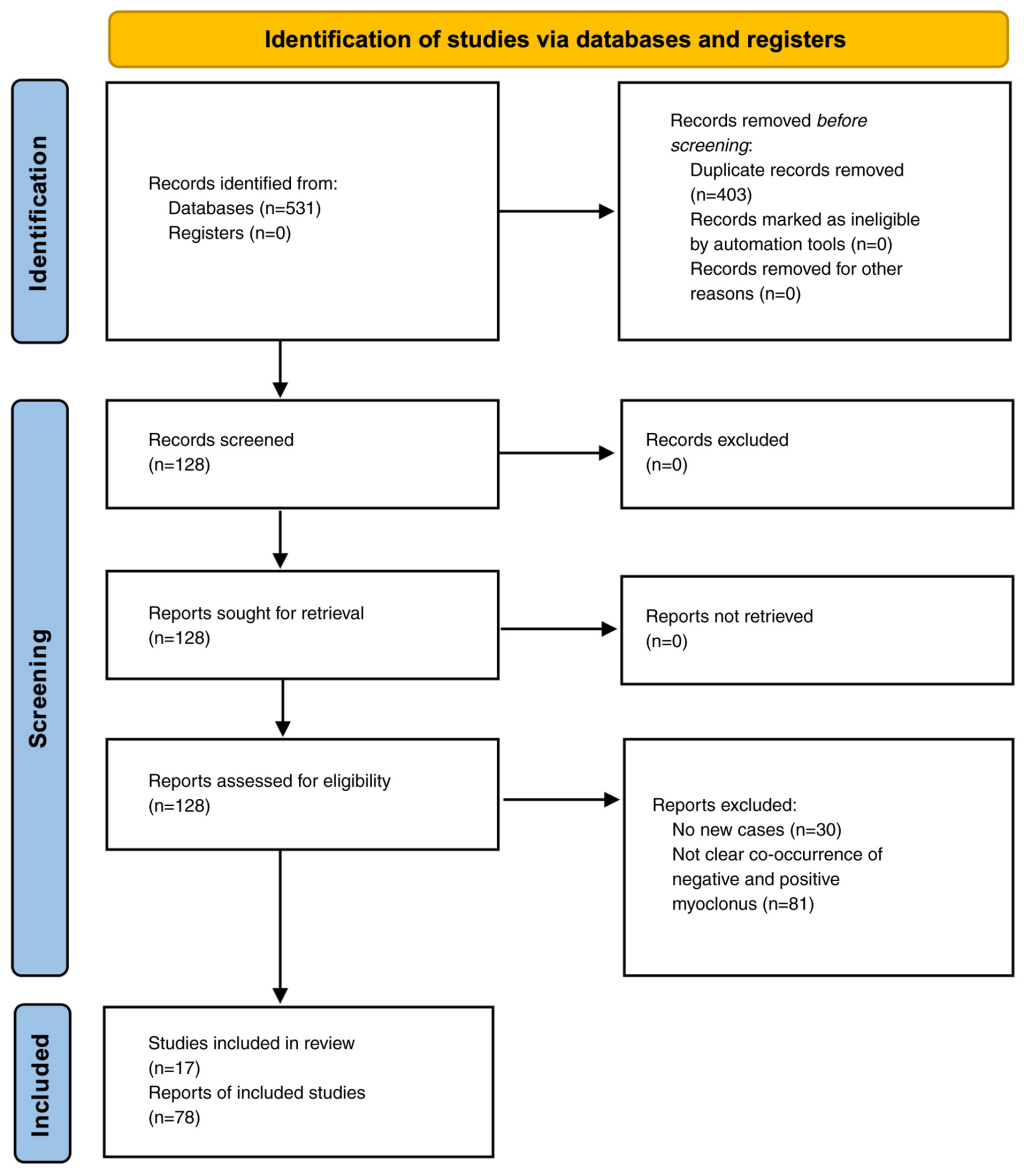
Flowchart of the study screening process for the present systematic review.

The present study was conducted in accordance with the Preferred Reporting Items for Systematic Reviews and Meta-Analyses (PRISMA) 2020 checklist, which provides a structured framework for systematically identifying, selecting, and synthesizing relevant studies (Fig. 1) (10). This approach ensures transparency, reproducibility and a comprehensive assessment of the available evidence. The assessment of the risk of bias in the included studies was evaluated with Joanna Briggs Institute (JBI) Critical Appraisal Checklist for Case Reports (11) (Table II) (1-3,7,8,12-22).

## Results

The present study found 17 reports containing 78 cases in the literature about TMS (Table III). Almost all the reports were from Japan, apart from the one by Sethi (21). The mean age of the patients was 75.67 years (standard deviation, 5.8 years), with a median of 75 years (range, 54-84 years). Sex was reported for 74 cases, and 60.8% of the individuals were male. A precipitating factor, such as an infectious disease or the introduction of a new medication, was observed in 24 cases (30.7%). All individuals achieved full recovery; however, 53 patients (67.9%) required benzodiazepine therapy, while the remaining individuals improved spontaneously.

Notably, chronic kidney disease was present in some cases of TMS. Reported creatinine levels ranged from 2 to 4 mg/dl (23). Notably, 29% (2 out of 7) of individuals described by Hashimoto *et al* (1) had stage 2-3 chronic kidney disease, and 1 patient in another study required dialysis (12).

The patient described by Kawakami (19) had a notable medical history of cardiac arrest. Electroencephalographic findings were non-specific, but may suggest hypoxic-ischemic brain injury, including cortical irritation, neuronal dysfunction, or epileptiform activity resulting from global hypoxia or ischemia.

A precipitating factor was identified in several cases of TMS. Cisplatin was reported in 1 case as a precipitating factor for the occurrence of the myoclonic jerks (1). Another patient received β-blocker and calcium channel blocker in therapeutic doses 24 h prior to the occurrence of myoclonic jerks. Initially, the authors considered drug-induced myoclonus as a potential differential diagnosis for TMS (14). Nevertheless, there was no occurrence of involuntary jerks after the reintroduction of these anti-hypertensive medications in the patients; thus, the drugs were unlikely to be related to the occurrence of TMS. Furthermore, the probability of TMS occurrence in drug-induced cases was also evaluated using the Naranjo algorithm (24), and these cases were categorized as doubtful.

Sethi (21) described the first case of TMS outside Japan, in a letter to the editor referencing the manuscript of Hiraga *et al* (7). Sethi (21) reported three cases with similar features that subsided spontaneously. However, Sethi (21) did not describe the phenomenology of the cases occurring with associated asterixis or the body localization. In addition, no details about ethnicity were provided. Based on the data from prior studies, the cases described by Sethi (21) lacked sufficient evidence to confirm a diagnosis of TMS. Moreover, the slowing shown in electroencephalograms was not related with encephalopathy in the cases described from Japan (25).

## Discussion

### Definition

TMS is characterized by an unusual type of involuntary jerk, which is recurring, involving mainly in the faciobrachial region. In addition, asterixis-like movement can occasionally be observed during these abnormal movements in 30-50% of the individuals with TMS (3). This abnormal involuntary movement was already described in the literature as ‘transient myoclonic state with asterixis (1)’, ‘benign transient shuddering-like involuntary movement (12)’ or ‘isolated transient myoclonus (7)’. Doden *et al* (3) defined that a single myoclonic burst of TMS has a duration of 44 msec (standard deviation, 12 msec) with a frequency of 9 hertz (standard deviation, 2 hertz). Ictal impairment and loss of consciousness are not observed following an episode of TMS; thus, the individual remains fully alert and is aware without experiencing any cognitive dysfunction or altered state of awareness during or after the episode.

Older populations with mild chronic systemic conditions are usually susceptible to TMS for unknown reasons. These patients are fully conscious, no disturbance of amnesia or paralysis is observed, and they responded to simple and complex commands. Movement or posturing leads to the aggravation of myoclonus, whereas sleep causes the jerk to settle and improve the myoclonus of the patient. The myoclonus exhibits no exacerbation by sensory stimuli. These jerks present acutely within a day and resolve spontaneously within a few days or respond well to treatment with benzodiazepines. Patients with TMS have exhibited a frequent recurrence of myoclonus and almost half the patients have experienced recurrence within a year and a half.

Periods of silence have been observed in electromyography (EMG) in patients with TMS. In fact, in the majority of cases, the brief period of silence has been observed during the refractory phases following positive myoclonic discharges, indicating a temporary cessation of neural activity between the bursts of involuntary muscle contractions (3). Ugawa *et al* (26) categorized the interruption of muscular activities into two types. The first type is the classically described form of asterixis, which is the silent period that follows an interval with no change in the background EMG activity, suggesting that muscle activity remained stable while the myoclonic discharges subsided. The second type of asterixis typically occurs after a sudden, involuntary muscle contraction triggered by voluntary movement or external stimuli. The technique known as ‘silent period locking’ involves precisely recording EMG during the silent period, allowing for the analysis of its association with preceding EEG activity. This technique has revealed that the EEG patterns prior to the silent period were specifically associated with the second type of asterixis silent periods, indicating a distinct neurophysiological connection between the brain’s electrical activity and the subsequent muscle inactivity (26). Some movement disorders specialists believe that asterixis associated with TMS should be classified as type II asterixis.

### Etiology

The etiology of TMS can vary widely, with no evidence of a definitive cause. Studies have shown a consistent occurrence of TMS in the older population. The majority of cases reported in previous studies are older males and older females, suggesting that aging can precipitate TMS (3). In addition, sex can likely be a predisposing factor, as it was observed that TMS occurs more frequently in males than females. The majority of these patients were found to have mild chronic comorbidities. Significant fluid status changes in patients with renal and cardiovascular disorder were likely associated with the occurrence of TMS (8).

**Figure 2:**
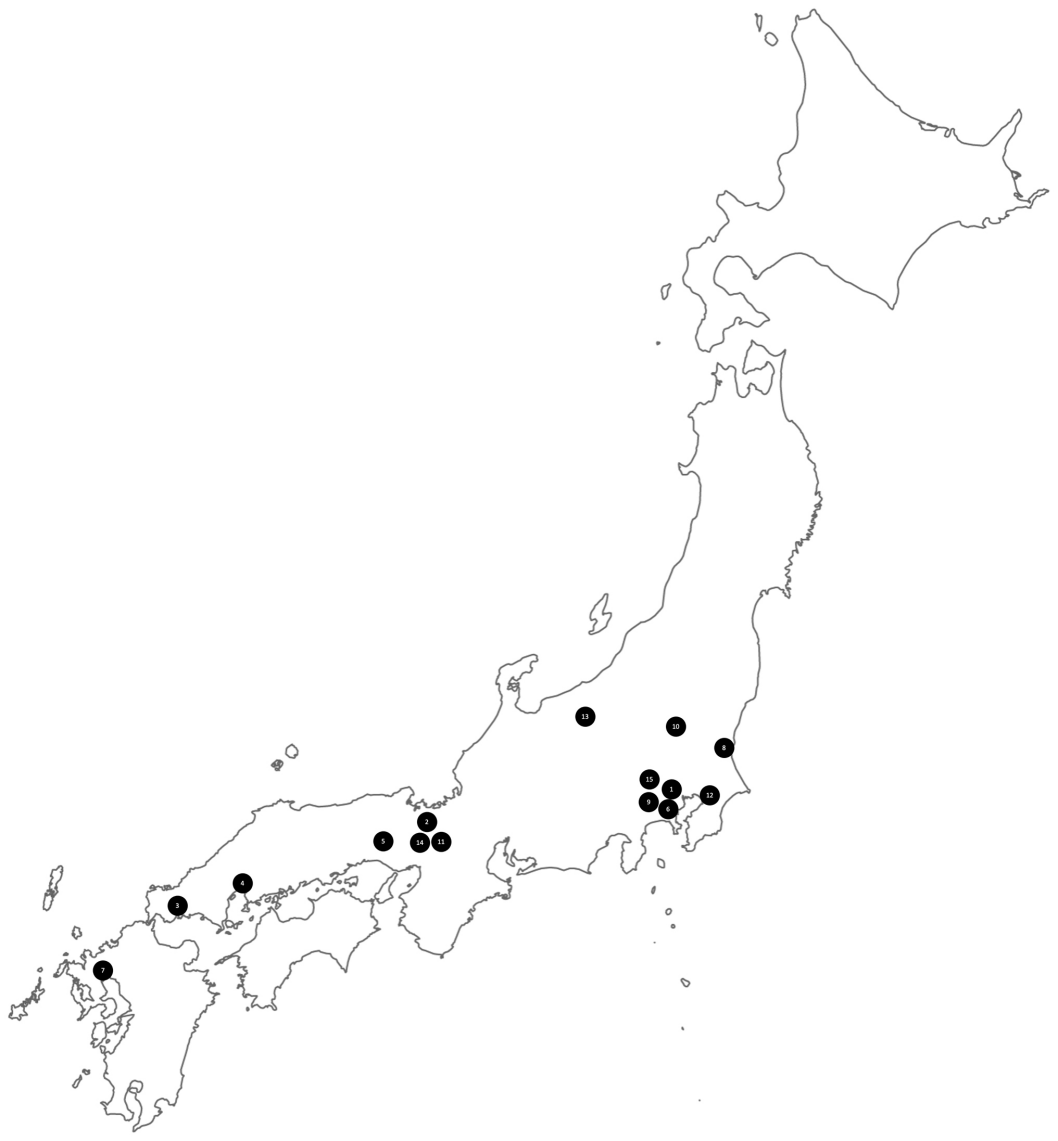
Geographical distribution in Japan of the cases reported in the literature of transient myoclonic state. The case described in the study by Sethi (21) was not included as it was outside Japan. In addition, there were insufficient data from the study by Tsuchiyama *et al* (23) to provide the geographical localization. The locations are indicated by numbers on the map and refer to the following studies: 1, Mizutani *et al* (2); 2, Hashimoto *et al* (1); 3, Negoro *et al* (12); 4, Fujihara *et al* (13); 5, Hirata *et al* (14); 6, Iijima *et al* (15); 7, Yamamoto (16); 8, Kohono *et al* (17); 9, Okuma *et al* (18); 10, Kawakami (19); 11, Hitomi *et al* (20); 12, Hiraga *et al* (7); 13, Doden *et al* (3); 14, Umemura *et al* (22); 15, Harada *et al* (8).

TMS is considered a spontaneous cortical myoclonus, with cortical hyperexcitability potentially indicating a genetic cause. However, the argument for a genetic basis is weak, as geographic clustering does not necessarily imply a genetic factor. This clustering could be attributed to environmental factors, underreporting in other regions, or publication bias. Notably, TMS cases have been reported exclusively in Japan, though there is no clear, specific geographical distribution within the country (Fig. 2).

A small number of TMS cases with increased levels of pyruvate and lactate were also reported; however, there is no definite evidence to prove this change of biochemistry in their body as a likely cause of TMS. This could have been a mere coincidence, and can be explained by continual muscle contractions (1). Another precipitating factor reported by patients in some studies is infection. Myoclonic jerks can occur in the initial, recovery, or the transition between these two phases, and this was observed in almost half of the patients with TMS (7). Elevated titer of Epstein-Barr virus IgG antibodies was also reported in some patients (2). Drugs such as cisplatin, risperidone and levofloxacin are among the other etiological factors causing myoclonic jerks. According to some studies, the initiation of the above-mentioned drugs has led to the onset of TMS due to its role in temporary metabolic changes and increased sympathetic nervous system activity (1,3). All the external etiological factors are likely to have caused TMS; however, a number of patients have no history of any event occurring prior to the onset of TMS. Thus, precipitating factors are not mandatory for TMS occurrence (20).

### Pathophysiology

TMS is characterized as a cortical myoclonus that is spontaneous and non-reflex (1). Variations in the physiological parameters with fluctuations in both brain electrical activity and muscle response have been observed. In electrophysiological studies, jerk-locked back averaging (JLA) revealed cortical activity before the occurrence of the involuntary muscle activity (18). Hitomi *et al* (20) reported that fluctuations in EMG signal variability were associated with the severity of myoclonus. In addition, increased amplitude or heightened response in the cortical area when sensory stimuli are applied [giant somatosensory evoked potentials (SEPs)] have been observed, although this is a rare phenomenon. This enlargement suggests heightened neural excitability or enhanced processing of sensory information within the brain’s sensory pathways.

Cortical spikes preceding myoclonic jerk, shown by JLA, suggests hyperexcitability present in primary motor cortex (PMC) during the myoclonic occurrence. The decrease on the amplitude of these positive spikes by the administration of benzodiazepines, resulted in the clinical improvement of the patient. This further suggests that selective activation at the PMC is likely the cause of involuntary jerks in TMS. Furthermore, the administration of benzodiazepines simultaneously suppresses the spikes in the PMC and myoclonus. In JLA, the spike that precedes the myoclonus has a positive polarity in the reflex myoclonus of cortical origin and a negative polarity in the spontaneous myoclonus (27). The negative polarity in JLA may result from a pathologically excitable cortex, similar to an epileptic state. Thus, a positive polarity suggests that TMS is a cortical myoclonus.

Motor-evoked potentials have been reported to be normal during the asymptomatic period, suggesting no hyperexcitability at the PMC. This suggests that cortical hyperexcitability was recorded only at PMC during the symptomatic phase, as shown by JLA, and it is associated with myoclonus in patients with TMS (3).

Other electrophysiological studies have demonstrated no giant SEPs during symptomatic or asymptomatic periods. SEP amplitudes tend to increase as a person ages; hence, the SEP amplitudes in TMS do not markedly increase (28,29). In addition, an overall amplitude increase does not necessarily indicate a giant SEP. EEG is usually normal, and there are no epileptiform discharges during the myoclonus, suggesting that an epileptic factor is unlikely to cause TMS (28).

The laboratory tests of patients with TMS were found to be within normal limits. Neuroimaging was unrevealing (7). Notably, areas of hyperperfusion in the bilateral precentral gyri during the myoclonic jerks were observed. This focal hyperperfusion persisted for three months in the precentral gyri even after the disappearance of the myoclonus. The hyperperfusion supports the hyperactivity in the PMC during the symptomatic period. It also indicates that hyperactivity can occur without presenting symptoms and contribute for TMS or its recurrence (22).

Subcortical structures may play a role in TMS. The brainstem and motor systems play a crucial role in the development of axial and bilateral myoclonus. The brainstem, which is responsible for basic autonomic and motor functions, coordinates movement and muscle control, while the motor systems involve pathways that regulate muscle tone and voluntary movements. When either of these systems is disrupted, it can lead to involuntary muscle jerks or twitches, particularly affecting the axial muscles (trunk and neck) and bilaterally (on both sides of the body) (1). Some authors have also reported the resemblance of TMS and shivering; thus, the hypothalamic region that is related to the thermal regulation may play a role in the development of this pathology (30). Of note, subcortical myoclonus cannot be excluded, as the cortical and subcortical regions can originate asterixis and positive myoclonus. In this manner, the cortical than the subcortical source is more likely to be related to the occurrence of TMS, although there is no clear definition and multiple sources from different locations in the brain may lead to this disorder (20).

Another hypothesis suggests that the excessive activation of specific areas of the brain may lead to TMS, while enhanced inhibitory effects could contribute to the development of negative myoclonus. Of note, a combination of negative and positive myoclonus has already been observed in multiple myoclonic syndromes such as post hypoxic myoclonus and myoclonic epilepsy syndromes (31).

### Diagnostic approach to transient myoclonic state. Initial assessment of patients with TMS

In terms of medical history, it is important to obtain the specific timeframe of the myoclonus onset, the progression of symptoms over time (acute vs. subacute/chronic), the body parts most commonly affected, alleviating and worsening factors, neurological family history and comorbidities (32). It is also important to determine whether symptoms are stable or progressive (32).

The categorization of the type of myoclonus largely depends on the age of onset. The onset of myoclonus in young adulthood, alongside with generalized epileptic seizures and neurocognitive impairment are suggestive of progressive myoclonus epilepsy (PME) (32). Moreover, in individuals who are > 65 years of age, myoclonus associated with cognitive impairment is frequently observed Lewy body dementia, corticobasal degeneration, in prion diseases and in advanced stages of Alzheimer’s disease (33). On the other hand, opsoclonus-myoclonus syndrome in childhood is most often associated with paraneoplastic disorders underlying malignancies, such as medulloblastoma and neuroblastoma (34). When manifesting in middle-aged adults, myoclonus can be part of paraneoplastic syndromes associated with skin and lung cancers. Myoclonus could also be secondary to drugs and other systemic conditions (35).

In terms of different timeframes of clinical manifestations, the acute onset of myoclonus is generally observed in hepatic and renal failure, as well as in other toxic-metabolic conditions, thyrotoxicosis, electrolyte abnormalities, hypoglycemia nonketotic hyperglycemia, and in central nervous system infections, such as herpes simplex encephalitis and neuroborreliosis (32). Myoclonus can also occur following hypoxic brain injury. The recent initiation of new medications or an increase in dosage should always be considered a potential cause of abnormal involuntary movements (35). Additionally, neurodegenerative diseases and PME are generally associated with myoclonus with a slow and progressive onset, worsening overtime (33).

It is worth mentioning that the presence of neurological findings apart from myoclonus may lead to algorithms assisting with the etiological diagnosis of involuntary movements. Metabolic disorders and even PME have a pattern of inheritance for genetic traits where a child inherits a mutated gene from each parent (32). On the other hand, dominant causes of myoclonic jerks involve non-neurodegenerative conditions like myoclonus-dystonia syndrome (33).

In the context of the neurological exam, it is essential to evaluate for the presence of postural myoclonus while the patient is keeping arms outstretched, at rest, and during movement (36). Spinal or brainstem sources are generally observed with myoclonus at rest, whereas myoclonus from cortical source is usually triggered by and generally manifests as focal distribution (37). Spinal segmental myoclonus is also considered focal, as it typically affects specific regions of the spinal cord and associated muscles, often localized to a particular segment or area (36).

Stimulus sensitivity analysis is another part of the neurological examination of myoclonus. Myoclonus can be triggered by gently touching the outstretched fingers, with the response being particularly sensitive to auditory stimuli (38). In this case, even subtle sounds may provoke involuntary muscle jerks, demonstrating the heightened responsiveness of the condition to auditory input. Hand-clapping may also induce myoclonus (38).

### Neurophysiology of cortical vs. subcortical myoclonus

The neurophysiology of myoclonus can be categorized based on whether it originates from cortical or subcortical regions, each with distinct mechanisms. Cortical myoclonus is typically associated with the motor cortex, which controls voluntary movement. The abnormal electrical activity arises from the cortex, often due to an issue in the pyramidal tract or cortical circuitry. It may occur in response to voluntary movement, sensory stimuli, or even spontaneously. Cortical myoclonus can be triggered by external stimuli (e.g., tactile, auditory) and is usually characterized by rapid, jerky muscle contractions. It is often seen in conditions such as epilepsy (e.g., juvenile myoclonic epilepsy) and neurodegenerative diseases (e.g., corticobasal degeneration). Subcortical myoclonus arises from deeper structures, such as the brainstem, basal ganglia, or spinal cord. It is typically less responsive to cortical stimuli and may result from dysfunction in the brainstem motor control centers or altered processing within the basal ganglia circuits. Subcortical myoclonus often involves more generalized or bilateral muscle jerks, which are not typically triggered by external stimuli. It is observed in conditions such as brainstem lesions, neurodegenerative diseases, or metabolic disorders. Both types of myoclonus result from abnormal neuronal firing but differ in their origin and the specific pathways involved (32).

### Differential diagnosis

Notably, non-epileptic myoclonic movements are more prevalent than their epileptic counterparts, particularly during in younger patients, such as infancy and childhood. Parents often report myoclonic movements, whether confirmed or not, making the differential diagnosis challenging. Various factors, including intellectual disabilities and EEG irregularities, can further complicate the diagnosis. Non-epileptic myoclonic movements can occur during the neonatal stage. One condition to consider in infancy is Fejerman myoclonus, which is considered to arise from a subcortical source. Myoclonus in this condition may be triggered by certain stimuli, such as touch or auditory cues, and can be part of a broader clinical presentation that includes other neurological signs (32).

It is important to differentiate Fejerman myoclonus from other similar conditions, such as benign neonatal myoclonus or early-onset myoclonus, before proceeding to genetic testing (39). Careful clinical evaluation is key to ruling out other forms of myoclonus. Research has indicated that conditions resembling myoclonus, such as head atonic episodes, often begin in the first months or years of life, with normal psychomotor development and a neurologically normal assessment (40). These conditions tend to spontaneously regress after a few months without long-term consequences. Misdiagnosis can have significant implications for both individuals and their families, as Fejerman myoclonus and head atonic episodes are frequently mistaken for epileptic seizures, leading to unnecessary treatments and emotional stress for parents and caregivers. Accurate diagnosis is critical to avoid mismanagement and ensure appropriate care (41).

Non-epileptic symptoms usually do not react to antiseizure medications (ASMs), leading to some children being wrongly diagnosed with epilepsy or even drug-resistant epilepsy. When the start of an ASM coincides with a natural reduction in episodes, this reaction can be inaccurately attributed to the ASM, resulting in the continuation of an unnecessary treatment for a number of years (42). Differentiating between epilepsy and other conditions can be effectively done through a more detailed history and by using videos taken by parents or recordings from video-polygraphy. Although neurophysiology can help determine the classification, it lacks specificity; therefore, it cannot provide the causes of the myoclonus (43).

There are some similarities between TMS and benign adult familial myoclonus epilepsy (BAFME) and familial cortical myoclonic tremor with epilepsy (FCMTE) (42). These conditions are relevant to discuss due to their overlap in clinical presentation, particularly in terms of myoclonus and tremor. However, TMS can be differentiated from BAFME and FCMTE by several key factors. These include the family history of the patient, which may reveal genetic predispositions; the age at which symptoms first appear, helping to understand the progression of the condition; the specific patterns of myoclonus experienced, which can vary in their distribution across the body; and the presence or absence of seizures, which may indicate different underlying mechanisms of the disorder. Each of these elements provides valuable insight into the nature of TMS and aids in its diagnosis and management (20).

### Future studies and specialist recommendations

Future studies on TMS are required to address several critical areas to enhance our understanding and treatment of this condition. First, research should focus on elucidating the neurobiological and genetic mechanisms underlying transient myoclonus, employing advanced neuroimaging techniques and molecular studies. Comprehensive clinical characterization through longitudinal studies is necessary to distinguish transient myoclonus from other movement disorders, identify subtypes, and develop standardized diagnostic criteria.

Epidemiological studies are vital for understanding the prevalence, incidence and risk factors associated with transient myoclonus. Population-based research and multicenter collaborations could provide insight into demographic and environmental factors, aiding in developing preventive strategies. Investigating potential genetic predispositions to TMS is critical, particularly in cases where there is a familial pattern. Further studies are warranted to identify genes linked to myoclonus and other movement disorders, which could provide insight into the etiology and lead to targeted therapies.

Further research is also required to investigate the impact of transient myoclonus on the quality of life and long-term outcomes of patients, using patient-reported outcome measures to assess treatment effectiveness. Research into the long-term prognosis of individuals with TMS is crucial. Further studies are required assess the likelihood of recurrence, the potential for neurodevelopmental delays, or any lasting neurological impairments. Finally, advances in genomics and biomarker discovery should be prioritized to identify diagnostic and prognostic markers, enabling early diagnosis and personalized treatment approaches. Collaborative, multidisciplinary efforts are crucial to advancing our understanding and improving care for transient myoclonus patients.

The present study reviewed all the particular features associated with TMS. An overview of the characteristics of TMS is presented in Table IV.

In conclusion, the present systematic review emphasizes the intricate nature of TMS or TMA. Future research endeavors are required to prioritize uncovering the biological and genetic mechanisms that contribute to this condition, as well as refining the criteria used for its diagnosis. Notably, TMS appears to have a distinct geographical preference, suggesting at a potential genetic distribution among affected populations. Understanding the pathophysiological mechanisms associated with this specific form of stereotyped myoclonus could provide valuable insight into other types of myoclonus as well. It is essential to engage in open discussions with patients and their caregivers about the typically benign progression of this disorder and to clarify that, in certain cases, the use of benzodiazepines may be necessary to manage symptoms effectively.

## Figures and Tables

**Table I:** Free text and medical subject heading search terms used in the US National Library of Medicine.

Query	Search term	Results
Transient myoclonus	(‘transiently’[All Fields] OR ‘transients and migrants’[MeSH Terms] OR (‘transients’[All Fields] AND ‘migrants’[All Fields]) OR ‘transients and migrants’[All Fields] OR ‘transient’[All Fields] OR ‘transients’[All Fields]) AND (‘myoclonus’[MeSH Terms] OR ‘myoclonus’[All Fields])	273
Transient myoclonus state	(‘transiently’[All Fields] OR ‘transients and migrants’[MeSH Terms] OR (‘transients’[All Fields] AND ‘migrants’[All Fields]) OR ‘transients and migrants’[All Fields] OR ‘transient’[All Fields] OR ‘transients’[All Fields]) AND (‘myoclonus’[MeSH Terms] OR ‘myoclonus’[All Fields]) AND (‘state’[All Fields] OR ‘states’[All Fields] OR ‘stated’[All Fields] OR ‘states’[All Fields] OR ‘stating’[All Fields])	40
Transient myoclonus state with asterixis	(‘transiently’[All Fields] OR ‘transients and migrants’[MeSH Terms] OR (‘transients’[All Fields] AND ‘migrants’[All Fields]) OR ‘transients and migrants’[All Fields] OR ‘transient’[All Fields] OR ‘transients’[All Fields]) AND (‘myoclonus’[MeSH Terms] OR ‘myoclonus’[All Fields]) AND (‘state’[All Fields] OR ‘states’[All Fields] OR ‘stated’[All Fields] OR ‘states’[All Fields] OR ‘stating’[All Fields]) AND (‘dyskinesias’[MeSH Terms] OR ‘dyskinesias’[All Fields] OR ‘asterixis’[All Fields])	16
Positive and negative myoclonus	(‘positive’[All Fields] OR ‘positively’[All Fields] OR ‘positiveness’[All Fields] OR ‘positives’[All Fields] OR ‘positivities’[All Fields] OR ‘positivity’[All Fields]) AND (‘negative’[All Fields] OR ‘negatively’[All Fields] OR ‘negatives’[All Fields] OR ‘negativities’[All Fields] OR ‘negativity’[All Fields]) AND (‘myoclonus’[MeSH Terms] OR ‘myoclonus’[All Fields])	202

MeSH, medical subject headings.

**Table II:** Quality evaluation of case reports by the Joanna Briggs Institute (JBI) Critical Appraisal Checklist for Case Reports.

Author(s), year of publication	Q1	Q2	Q3	Q4	Q5	Q6	Q7	Q8	(Refs.)
Mizutani *et al*, 1986	Y	Y	Y	Y	Y	U	Y	N	(2)
Hashimoto *et al*, 1992	Y	Y	Y	Y	Y	Y	Y	N	(1)
Negoro *et al*, 1994	Y	Y	Y	Y	Y	Y	Y	N	(12)
Fujihara *et al*, 1995	Y	Y	Y	Y	Y	Y	Y	N	(13)
Tsuchiyama *et al*, 1995	U	U	U	U	U	U	U	N	(23)
Hirata *et al*, 1997	Y	Y	Y	Y	Y	Y	Y	N	(14)
Iijima *et al*, 1997	Y	Y	Y	Y	Y	U	Y	N	(15)
Yamamoto, 1997	Y	Y	Y	Y	Y	U	Y	N	(16)
Kohono *et al*, 1998	Y	Y	Y	Y	Y	Y	Y	N	(17)
Okuma *et al*, 1999	Y	Y	Y	Y	Y	Y	Y	N	(18)
Kawakami, 2007	N	N	N	N	Y	Y	U	N	(19)
Hitomi *et al*, 2011	Y	Y	Y	Y	Y	Y	Y	N	(20)
Hiraga *et al*, 2014	Y	Y	Y	Y	Y	Y	Y	N	(7)
Sethi, 2014	N	N	N	N	Y	U	Y	N	(21)
Doden *et al*, 2015	Y	Y	Y	Y	Y	Y	Y	Y	(3)
Umemura *et al*, 2015	Y	Y	N	U	Y	Y	Y	N	(22)
Harada *et al*, 2024	Y	N	N	N	Y	Y	Y	N	(8)

The questions in the checklist are as follows: Q1, were patient’s demographic characteristics clearly described? Q2, was the patient’s history clearly described and presented as a timeline? Q3, was the current clinical condition of the patient on presentation clearly described? Q4, were diagnostic tests or assessment methods and the results clearly described? Q5, was the intervention(s) or treatment procedure(s) clearly described? Q6, was the post-intervention clinical condition clearly described? Q7, were adverse events (harms) or unanticipated events identified and described? Q8, does the case report provide takeaway lessons? N, no; U, unclear; Y, yes.

**Table III:** Literature review of reports of transient myoclonic state.

Author(s), year of publication	Prefecture, country	No. of cases	Age (years)/sex	Clinical manifestations	Precipitator	Recovery	Management	(Refs.)
Mizutani *et al*, 1986	Tokyo, Japan	1	84/M	Lasted 2-7 days. Recurred five times in 2 years. Viral capsid antigen IgG antibodies for Epstein-Barr virus were positive.	1 IF	1 SP	None	(2)
Hashimoto *et al*, 1992	Kyoto, Japan	7	72.3 (mean)/ 3 F and 4 M	Recurrent.	1 IF, 1 drug	7 BZD	IM/IV DZP	(1)
Negoro *et al*, 1994	Yamaguchi, Japan	9	70.5 (mean)/ 4 F and 5 M	Lasted 0.5-4 days. Recurrent.	3 IF	3 SP and 6 BZD	PO CZP	(12)
Fujihara *et al*, 1995	Hiroshima, Japan	1	76/M	Recurrent.	0 IF	1 BZD	PO CZP	(13)
Tsuchiyama *et al*, 1995	Japan	1	NA	NA	NA	1 BZD	NA	(23)
Hirata *et al*, 1997	Hyogo, Japan	1	54/F	No recurrence.	0 IF	1 BZD	PO CZP and IM/IV DZP	(14)
Iijima *et al*, 1997	Tokyo, Japan	1	84/F	No recurrence.	0 IF	1 SP	None	(15)
Yamamoto, 1997	Saga, Japan	2	67.5 (mean)/ 1 F and 1 M	Elevated serum amyloid A protein. Recurrent.	1 IF	2 SP	None	(16)
Kohono *et al*, 1998	Ibaraki, Japan	2	60.5 (mean)/ 2 M	Recurrent.	1 IF	1 BNZ and 1 SP	PO CZP	(17)
Okuma *et al*, 1999	Tokyo, Japan	5	71.5 (mean)/ 1 F and 4 M	No recurrence.	1 IF	3 SP and 2 BZD	PO CZP, IV/IM DZP	(18)
Kawakami, 2007	Tochigi, Japan	NA	NA	NA	NA	NA	NA	(19)
Hitomi *et al*, 2011	Kyoto, Japan	6	84 (mean)/ 2 F and 4 M	There was no increase in SEP amplitudes during symptoms, and JLA showed a positive spike occurring before the myoclonic jerks.	1 IF	6 BZD	PO CZP, IM/IV DZP	(20)
Hiraga *et al*, 2014	Chiba, Japan	11	75 (mean)/ 5 F and 6 M	Lasted 1-4 days.	5 IF	7 SP and 4 BZD DZP	PO CZP, IM/IV	(7)
Sethi, 2014	USA	3	NA	NA	NA	3 SP	None	(21)
Doden *et al*, 2015	Nagano, Japan	26	79.7 (mean)/ 10 F and 16 M	Lasted days.	9 IF	22 BZD and 4 SP	PO CZP, IV/IM DZP	(3)
Umemura *et al*, 2015	Kyoto, Japan	1	79/ M	Iodine-123 iodoamphetamine revealed increased perfusion in the bilateral primary motor cortex.	NA	1 BZD	PO CZP	(22)
Harada *et al*, 2024	Tokyo, Japan	1	68/ F	NA	NA	1 BZD	PO CZP and IV/IM DZP	(8)

BZD, benzodiazepine; CZP, clonazepam; DZP, diazepam; F, female; IF, infection; IM, intramuscular; IV, intravenous; JLA, jerk-locked back averaging; M, male; NA, not available/ not applicable; PO, per os; SEP, somatosensory evoked potentials; SP, spontaneous.

**Table IV:** Transient myoclonic state features (as indicated in the manuscript).

1. Older individuals and those with chronic illnesses are vulnerable.
2. The state of consciousness is almost normal or just slightly compromised.
3. Myoclonic jerks happen unexpectedly and are somewhat intensified during movement, primarily observed in the fasciobrachial region.
4. Voice can be tremulous. However, no opsoclonus or palatal myoclonus are observed.
5. No noticeable myoclonic jerks are triggered by sensory stimuli.
6. Negative myoclonus is present in the muscles affected by myoclonic jerks, and similar movements resembling negative myoclonus can also be observed when the tongue is extended.
7. Neurological examination apart from myoclonus is within normal limits.
8. The electroencephalogram reveals nonspecific slowing or irregular patterns, with no evidence of epileptiform abnormalities. Photoparoxysmal response is not observed.
9. The beginning of this condition is relatively sudden, but it slowly became more noticeable over the course of several hours.
10. Myoclonus and asterixis disappear altogether within three days of onset with benzodiazepine therapy.
11. No lasting effects were observed, although the transient myoclonic state has a tendency to recur.
12. Electrophysiological results indicate the absence of giant somatosensory evoked potentials during both symptomatic and asymptomatic phases, as well as a lack of cortical reflex. A positive spike in jerk-locked back averaging is observed prior to the myoclonus. Motor evoked potential findings are normal during an asymptomatic phase.

## Data Availability

The data generated in the present study may be requested from the corresponding author.
